# Integrated Models for Solid Waste Management in Tourism Regions: Langkawi Island, Malaysia

**DOI:** 10.1155/2011/709549

**Published:** 2011-09-04

**Authors:** Elmira Shamshiry, Behzad Nadi, Mazlin Bin Mokhtar, Ibrahim Komoo, Halimaton Saadiah Hashim, Nadzri Yahaya

**Affiliations:** ^1^Institute for Environment and Development (LESTARI), University of Kebangsaan Malaysia (UKM), Malaysia; ^2^Southeast Asia Disaster Prevention Research Institute (SEADPRI), University of Kebangsaan Malaysia, 43600 Bangi, Malaysia; ^3^National Solid Waste Management Department, Ministry Of Housing and Local Government, 50782 Kuala Lumpur, Malaysia

## Abstract

The population growth, changing consumption patterns, and rapid urbanization contribute significantly to the growing volumes of solid waste that are generated in urban settings. As the rate of urbanization increases, demand on the services of solid waste management increases. The rapid urban growth in Langkawi Island, Malaysia, combined with the increasing rates of solid waste production has provided evidence that the traditional solid waste management practices, particularly the methods of waste collection and disposal, are inefficient and quite nonsustainable. Accordingly, municipal managers and planners in Langkawi need to look for and adopt a model for solid waste management that emphasizes an efficient and sustainable management of solid wastes in Langkawi Island. This study presents the current practices of solid waste management in Langkawi Island, describes the composition of the solid waste generated in that area, and presents views of local residents and tourist on issues related to solid waste management like the aesthetic value of the island environment. The most important issue of this paper is that it is the first time that integrated solid waste management is investigated in the Langkawi Island.

## 1. Introduction

The Malaysia tourist industry rapidly is expanding because of increasing international and domestic tourists. Generation of waste in per capita of Langkawi Island is more than in compare with Malaysia (according to our research and project). The natural characteristics of Langkawi local ecosystems with coral reefs, sand beaches, and mangroves in Geopark are affected under threat by poorly waste management and cause other environmental problems.

Knowledge about solid waste sources and types as well as information on its composition and rates of production and disposal is essential for the design and operational facets of the functional elements concomitant to solid waste management. On the other side, solid waste may be classified on the foundations of generation source and type [1]. Source-based classification of solid wastes classifies the wastes on the basis of their origins. Thus, this classification perspective is vital for the waste management process as it provides information related to the origin of the waste which will eventually help in managing the waste at its source of release [2]. The classification types of categorized wastes by consideration to their nature and produced source are wide-ranging and significant in fact. Knowledge of the nature of the wastes is crucial for the waste management process since it can help waste managers deal with the different types of wastes in appropriate ways as well as reduce the potential negative impacts attendant to its waste handling and handlers [3]. 

Worldwide, one of the most dynamic economic activities is tourism. Over the last few decades tourism has developed spectrally and become global industry and one of the fastest growing trades [4]. It is regarded as an integral component of Langkawi Island and its costal economy [5]. Year after year, further attractions have evolved on the grounds of the potential and facilities of trips to adventurous areas, diving, boat-trips, and observation of wildlife (e.g., birds, dolphins, and corals) in the island and these possibilities have made coastal destinations far more attractive [4, 5]. 

Tourism plays a substantial role in the development of a small island like Langkawi. Yet, it results in creation of additional wastes which have indirect and direct impacts on the somewhat unique and quite vulnerable environmental resources. In light of this, an identification of the negative and positive effects of the growing tourism industry in Langkawi Island as well as evaluation of the net balance between these is an objective that is necessary for development of a sustainable tourism industry in the area. It is well established that development of the global tourism industry in islands in the recent years has led to increased production of wastes and to altering the composition of these wastes as well. Securing sustainable tourism industry in islands should set reducing resource consumption and waste generation as a basic principle and priority. Reducing overconsumption and the amounts of waste produced has two dimensions: conserving the resources utilized by the tourists to be employed in production of different goods and releasing pressure on environmental resources that result from treatment and disposal of wastes. As far as waste production and management are concerned, what is really important is confirming dominance of sustainable waste management patterns rather than the amounts of waste which are produced.

Malaysia is spending 75 percent of municipality budget for waste collection. According to the available statistics, otherwise in Langkawi Island there is a problem of collection and transportation to landfill. The biggest challenges are related to the municipalities for services of sustain waste management in a sound and effective manner.

Tourists generate substantial amounts of solid waste. It has been estimated that tourists generate double solid waste per capita compared to local residents in Langkawi Island. They also generate substantial amounts of liquid waste, much of which goes untreated.

These problems have been exacerbated by the small size, remoteness, and rapid urbanisation of Langkawi islands. Contaminated environment is an increasing population pressures consequence. The household waste majority of is recyclable material and organic waste. Unfortunately, amount of recyclable material is few and no real and good market for recyclable material. Waste also is generally burned or dumped in some areas near roads and waste pollutants are increasing [6]. 

Consequently, waste management is a critical challenge of Geopark environment in the region. Waste pollution and illegal dumping will have impact on people health, potential of tourism, Geopark characteristic, and sustainable development in the island.

The tourism industry can have both positive and negative impacts on tourist destinations, such as Bali, Indonesia, and Thailand [7]. Benefits of tourist industry include economic opportunities and employment creating. Negative impacts of tourist industry in Langkawi Geopark include natural resources deterioration and problems of increasing solid waste quantities.

The negative impacts of tourism in the area include natural resources consumption, consumerism, and generation of waste. Hotels often generate large quantities of solid waste because tourists use the materials in the small packaging forms. Improper management of waste causes environmental degradation and aesthetic appeal losing, in shape of litter on beaches and streets, illegal dumping, and garbage burning.

Sustainable SWM principles include equity (for all citizens that are entitled to an appropriate waste management system due to environmental health reasons, promote the health issues for resident and tourist, and minimise the waste production for resident in island), effectiveness (related to safe removal of waste management, protection of environmental quality and sustainability, and maximing 3R), and efficiency and sustainability of solid waste management related to increasing benefits and decreasing of costs. 

The planning program of solid waste management optimization will have an important impact when the government plays the role of the facilitator and catalyser to implement partnerships between stakeholders. The municipality should encourage the waste management development with utilising waste separation at source, waste reduction, reuse, recycling, and composting. As the facilitator (using concepts such as the polluter pays principle and cleaner production for development of waste management program), the government should protect the Langkawi Geopark and support the society, funding, training, technical assistance, information exchange, and monitoring.

The key elements for a successful ISWM implementation program in hotels are management's initiative and training of personnel [8]. For successful ISWM program, it is necessary for hotel management to be convinced of improved waste management importance.

With the top management support, the necessary resources can allocate for improvement process. Improve environmental quality (with the long-term profit to preserve the attractive region) or even generate additional income through the recyclables selling or compost industry due to using enrich soil. Continuous capacity building of hotel staff is needed. Staff contacts with waste are in the lower organization parts mostly. Rates of Rotation and attrition at this special step are high extremely. Also programs of capacity building for staff should be arranged.

Improper SWM can lead to contaminant and deterioration of the aesthetic appeal of tourist destinations. Integrated solid waste management (ISWM) may be defined as selection and implementation of appropriate techniques, technologies, and management programs to realize certain waste management goals and objectives. The hierarchy of integrated solid waste management of the United States Environmental Protection Agency (USEPA) follows the priority order: source reduction, recycling, waste combustion or waste transformation and land filling [9]. 

## 2. Study Area

Langkawi Island comprises archipelago of 99 islands located in the Andaman Sea, around 30 km off the mainland coast of northwestern Malaysia. Pulau Langkawi has a population of approximately 96,726. The total land area of the islands is 47,848 hectares while Langkawi, which is the main island, has an area of 32,000 hectares. From north to south, Langkawi is nearly 25 km long and from east to west it is almost as wide. The coastal areas consist of flat alluvial plains with limestone ridges. Two-thirds, approximately, of the island are predominated by natural vegetation, hills, and forest-covered mountains. The island is characterized by a sunny, hot, and humid tropical climate. The average annual temperature is about 32°C (33–24°C), and the average yearly rainfall is around 2500 mm. The rainy season extends from August to September. 

 The landfill site in Langkawi Island is known as Tapak Pelupusan Sisa Pepejal Kampung Belanga Pecah. It has been in service since 1985 with daily solid waste input of 80 tons on the average. Only 10 years of the lifespan of this land-filling site are yet left. Landfill in the area is not sanitary and leachate with run-off near landfill is valid specially in high raining season. Separation of waste has not been at source; it is one of the waste issues need to discuss and educate the people (Table 1).

## 3. Methodology

In this study we used American Society for Testing Materials (ASTM) that Field sampling was implemented during one week, whereby a total of 35 samples were taken (five samples per days).

Hitherto, there are no standard procedures adopted by the Government of Malaysia (GOM) for MSW characterization study. As such, procedures outlined in ASTM will be used as a guide in carrying out all the sampling and laboratory works. 

In the study area was gathered randomly from a list of 35 ward names written on pieces of folded paper and placed in a container. Picking was done through a non-replacing method to prevent picking the same ward more than once. Subsequently, this was followed by sampling a number of households following the systematic random sampling approach. One individual from each sample household was interviewed for data collection purposes. Additionally, a questionnaire prepared and provided to the residents of Langkawi Island as it was deemed that feedback from which will (i) elucidate the local's level of awareness of issues related to solid waste and solid waste management and (ii) contribute to an understanding of issues related to solid waste management such as the approaches employed, the limitations to sustainable waste management, management needs, amongst others. 

**Figure 1:**
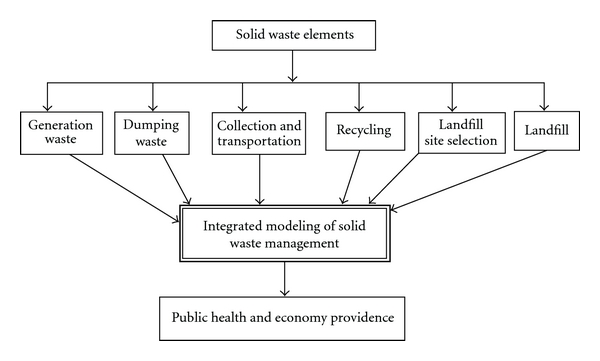
Structure of integrated solid waste management in Langkawi Island.

Waste management systems in Langkawi Island are under pressure because of increasing population and urbanization, changing patterns of consumption, tourism industry. Economic costs of solid waste will be very large in Langkawi Island if all significant variables and also optimization of the collection and transportation not be considered (Figure 1).

## 4. Result and Discussion

**Figure 2:**
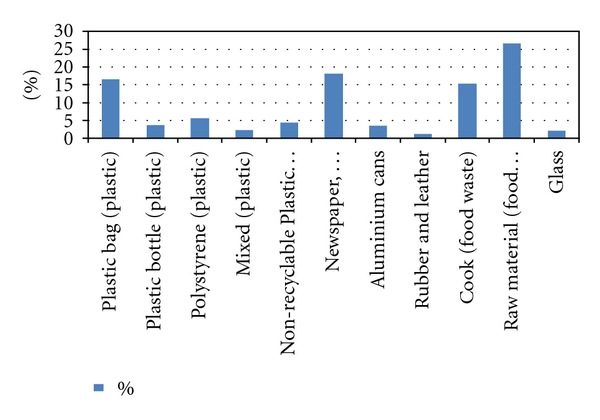
Mean composition of the waste samples from Langkawi Island.

The results of this study indicated that raw material has the highest percentage of the solid waste generated in the island. Raw materials and food waste was ranked and comprised 26.68% of the solid waste. Paper and then plastic bag wastes ranked the second and third highest group (18.15% and 17.04%) (Figure 2).

Feedback on the questionnaire showed that the municipality of Langkawi has been facing numerous obstacles and challenges associated with solid waste management that prevented it from addressing the challenges of solid waste management in the island in a proper and efficient manner. 

The problems attendant to poor management of solid wastes in relation to tourists, besides other related issues that were highlighted by the respondents, call for urgent attention of the municipal authority in Langkawi. Around 34.8% of the respondents indicated that improper management of the solid waste will contribute to increased environmental problems and may lead to closing the Kilim River branch of the Geopark, while 23.2%, 12.4%, 16.4%, and 13.2% of the total respondents expressed that poor solid waste management will lead to limited tourist visits, high levels of soil and water pollution, degraded aesthetic value of the environment, and steady objectionable odor release, respectively. These results agree with earlier findings of [10, 11].

Potential efficiency between sustainable development of Tourist and solid waste management in Island is including cooperation between sustainable tourist development in Langkawi island and integrated solid waste management, both of them affect on policy alternatives, in addition to role of stakeholders in economic condition, social and environmental impacts on solid waste management.

Part of the questionnaire aimed at drawing possible suggestions on how waste-related laws and regulations can be enacted. Almost 55.3% of the respondents supported that waste-related laws can be made more effective through public sensitization of the existing laws while 32.9% argued that these laws may be made effective via proper enforcement by the related law enforcement agencies. In addition, 14.5% of the respondents suggested that the waste legislation can be made more effective by means of instant fine posed on the offenders while only few respondents (2.9%) supported that such legislation may be made more effective by proper, regular monitoring.

Improvements in the solid waste collection services will to some degree contribute positively to reducing the amounts of waste in and around the municipality area. As the questionnaire showed, about 35% of the respondents claimed that public awareness on solid waste collection issues is vital for the municipality to manage the solid waste. On the other hand, nearly 37 % of the respondents highlighted that there is a need to engage the public in managing the solid waste. Moreover, almost 20.2% of the respondents supported that enforcing the waste and waste management legislations is important for dealing with the wastes in the municipality while 7.8% suggested that charging some fee, or pay as you pollute, that is, implementation of the “polluters pay” principle, may play a positive role in controlling the generation of waste by the public and subsequently minimize the costs of management, particularly the costs of waste collection.

Groundwater Pollution because of high level, surface water pollution from land based sources such as domestic sewage should be mentioned. They cause to carry risks for human health and can degrade habitats such as coral reefs, and tourist attractions such as beaches. The management of tourist should be attention to left of eagle feed at Geopark that oily waste cause to produce problem for mangrove trees in area. In island the facilities are inadequate either because a trained manpower shortage; consequently, poorly-treated effluent of waste leachate is often discharged into the branches of Geopark that is near the landfill site environment.

The problem in regulations of Langkawi Island has not been very effective according to questionnaire because inadequate institutional and human resource capacities to enforce them. Insufficient Facilities to storage and disposal of hazardous wastes is the problem too in Langkawi Island.

## 5. Conclusion

The present system of solid waste management cannot meet growing volumes of solid wastes that are produced daily. The central government should find legal and institutional framework for municipal solid waste management (MSWM) and guarantee that local governments have the authorities, powers, and capacities necessary for effective management of the solid waste. Enhancing the solid waste management practices in Langkawi island, the municipality needs to establish waste management strategies and employ the technological innovations due for securing improved waste separation at the source, resource recovery or recycling, and sanitary disposal of the solid wastes. 

While examining the local level of awareness on the benefits of appropriate solid waste management system to the local community, the researcher noticed that the residents of Langkawi Island were quite aware of the value of a clean and waste-free environment in terms of environmental health and safety, aesthetic value of the island, encouraged tourism, and reduction in spread of vector diseases. Inadequate and/or inappropriate management of the solid waste will enhance survival, growth, and spread of vectors which will hence endanger the people and environment besides polluting the water, soil, and air with organic and inorganic materials. It is much likely that varying proportions of the waste could be washed to the surface water bodies and consequently aggravate the environmental pollution problems. New strategies can improve tourism industry in the area without serious environmental problems and minimum dangerous effect on the natural Geopark.

## 6. Recommendations

We recommend establish the working relationships among hotels, resorts, and companies of waste collection. We also suggest good practices of environment, the most significant of them is developing integrated solid waste management (ISWM) approach at the hotel level.

Successful programs of ISWM at the hotel level need necessary elements such as initiative of management, adequate space for the separation of the waste, and activities of treatment related to capacity building plan for hotel personnel.

Usually tourist visits only short periods of time -one to two weeks- in the Langkawi Island which poses a challenge for awareness raising purposes between tourists and residents; as a result expanding separation at source will be useful at hotel level in island and education to public participation for separation at source.

Achieving a better services provision to improve infrastructure is effective by relationship establishment between private and public centres in Langkawi Island.

Lack of local recycling capacities adds to waste problem.

## Figures and Tables

**Table 1:** Population of tourism in Langkawi (2006–2009).

Years	2006	Changes percent	2007	Changes percent	2008	Changes percent	2009 until September
Population	981646	2.88	3426426	14.92	2879270	15.97	1044163

## References

[1] Nadi B, Mahmud AR, Shariff AR, Ahmad N (2009). Use of geospatial technology for landfill site selection. *Journal of Environmental Science and Engineering*.

[2] Ramachandra TV (2006). *Management of Solid Waste*.

[3] Agamuthu P (2001). *Solid Waste: Principles and Management: with Malaysian Case Studies*.

[4] CoastLearn (2009). *Sustainable Tourism. Introduction to Coastal Tourism*.

[5] Davenport J, Davenport JL (2005). The impact of tourism and personal leisure transport on coastal environments: a review. *Estuarine, Coastal and Shelf Science*.

[6] Nadi B, Mahmud AR, Ahmad N (2010). Managing of urban solid waste by geoinformatics technology. *International Geoinformatics Research and Development Journal*.

[7] Tribe J, Font X, Griffiths N, Vickery R, Yale K (2000). *Environmental Management for Rural Tourism and Recreation*.

[8] Shamshiry E, Nadi B, Mokhtar MB, Komoo I, Hashim HS (2011). Urban solid waste management based on geoinformatics technology. *Journal of Public Health and Epidemiology*.

[9] Tchobanoglous G, Kreith F (2002 & 2007). *Handbook of solid waste management*.

[10] Skordilis A (2004). Modelling of integrated solid waste management systems in an island. *Resources, Conservation and Recycling *.

[11] Chen MC, Ruijs A, Wesseler J (2005). Solid waste management on small islands: The case of Green Island, Taiwan. *Resources, Conservation and Recycling *.

